# DEVELOPMENT OF ORTHOPEDIC SIMULATOR TO PRACTICE CLOSED REDUCTION OF PEDIATRIC FRACTURES OF THE MIDDLE THIRD OF FOREARM

**DOI:** 10.1590/1413-785220233101e252387

**Published:** 2023-02-20

**Authors:** JAMIL SONI, LUIZE KREMER GAMBA, FABIANA DE SOUZA BEBBER, FRANCISCO KOZOVITS

**Affiliations:** 1 Pontifícia Universidade Católica do Paraná, School of Medicine, Curitiba, PR, Brasil.; 2 Hospital Universitário Cajuru, Curitiba, PR, Brasil.

**Keywords:** Simulation Training. Forearm Injuries. Closed Fracture Reduction. Education, Medical, Treinamento por Simulação, Traumatismos do Antebraço, Redução Fechada, Educação Médica

## Abstract

In the current scenario of medical education, a trend of using models and simulators to train operational skills, especially in the practice of basic orthopedic techniques, is growing. This form of teaching allows academics to maximize learning opportunities and contributes to improving the quality of care for their future patients. However, the realistic simulation has high costs as a major limitation.

**Objective:**

To develop a low-cost orthopedic simulator for practicing pediatric forearm reduction skills in the preclinical setting.

**Methods:**

A model of an arm and forearm with a fracture in the middle third was developed. Orthopedists, residents, and medical students evaluated the simulator’s ability to reproduce fracture reduction.

**Results:**

The simulator had a significantly lower cost than the others in the literature. The participants agreed that the model had a good performance, and that the manipulation was consistent with the reality of reducing closed pediatric forearm fracture.

**Conclusion:**

The results suggest that this model can be used to teach orthopedic residents and medical students the skill of closed reduction of fractures in the middle third of the forearm. ***Level of Evidence III, Case Control Study.***

## RESUMO

No cenário atual de ensino médico existe uma tendência crescente do uso de modelos e simuladores para o treino de habilidades operacionais, principalmente na prática de técnicas ortopédicas básicas, que permite aos acadêmicos maximizarem as oportunidades de aprendizado e contribui para melhorar a qualidade de atendimento dos futuros pacientes atendidos. A simulação realística, no entanto, tem como grande limitação os altos custos.

**Objetivo:**

Desenvolver um simulador ortopédico de baixo custo para a prática de habilidades de redução incruenta do antebraço pediátrico no cenário pré-clínico.

**Métodos:**

Desenvolveu-se um modelo de braço e antebraço com fratura no terço médio, que foi avaliado por médicos ortopedistas, residentes e acadêmicos de medicina quanto à capacidade do simulador de reproduzir a redução da fratura.

**Resultados:**

O simulador desenvolvido teve custo significativamente inferior aos existentes na literatura. Os participantes concordaram que o modelo teve um bom desempenho e que a manipulação foi condizente com a realidade de redução de fratura incruenta do antebraço pediátrico.

**Conclusão:**

Os resultados levam a crer que esse modelo pode ser usado para ensinar a redução incruenta de fratura no terço médio do antebraço para residentes de ortopedia e acadêmicos de medicina. **Nível de Evidência III, Estudo de Caso-Controle.**

## INTRODUCTION

Medical education aims to provide the best training possible for students at a reasonable operational cost. Students should be able to repeat technical activities or clinical procedures as many times as necessary to adequately develop their skills. However, this requires patient exposure and high costs.^1^ Alternatively, realistic simulation provides a controlled, safe, error-tolerant teaching environment for training of medical skills.^2^

The use of orthopedic simulators, even during medical school and residency, seeks to prepare health professionals to be able to identify, manipulate, and/or immobilize a patient with a fracture, and refer the patient to a specialized service where they will receive the final treatment. With an adequate initial care, the patient will benefit from reduced pain, reduced risk of compartment syndrome, and other complications, as well as greater comfort when moving.

In pediatric orthopedics, mastering basic orthopedic techniques for closed reduction of fractures is especially important. This is because, unlike in adults, conservative procedures are considered the best therapeutic option in many situations due to the specific bone characteristics of pediatric patients.^3^

Among the most common childhood traumas, 45% are represented by forearm fractures, with 80% of the time involving the ulna and radius.^3^ In these cases, the goal of treatment is to achieve fracture healing in good position and maintain normal range of motion of the elbow, wrist, and prono-supination. Bone remodeling allows the acceptance of certain deviations, and the adequate closed reduction maintained with cast immobilization is the best therapeutic option.^4^

In view of the social and epidemiological importance of forearm fractures in children, this study aimed to develop a device that portrays the clinical practice necessary for students and residents to practice closed reduction of these types of fractures.

In the literature reviewed, three relevant forearm fracture closed reduction simulators were found, all of them of distal fractures, two pediatric and one adult. The cost of these simulators ranged from 175 to 455 US dollars.^5^ ^)-(^ ^7^

### Objective

To develop a low-cost orthopedic simulator for teaching and training closed reduction of pediatric fractures during undergraduate and residency training.

## MATERIALS AND METHODS

For the technical development, the interface chosen for the construction of the simulator was that of a pediatric arm and forearm, compatible with an eight-year-old child with a fracture in the middle third of the forearm. The external structure and bones were made by Pacific Research Lab - Sawbones^®^. The structure consists of a silicone and polyurethane envelope that mimics skin and soft tissue. Radiolucent bones (humerus, ulna, and radius) with the presence of articular movements were used.

To stabilize the simulator during reduction practice, the elbow joint was fixed using a metal plate at a right angle, whereas the ulna and radius were distally fixed using plastic rods connecting the radius and ulna to the first and fifth fingertip, respectively. The use of screws for fixation was discarded due to the friable nature of the synthetic bone material, and the materials were attached to the bones using 0.9 mm stainless steel wires.

**Figure 1:**
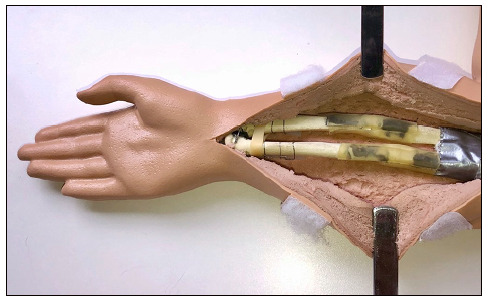
The stress system on the fracture seen through the anterior median opening of the simulator.

To mimic the tension forces exerted by the thick periosteum and soft parts on the drill, 1.25-mm thick latex strips were used. Three five-centimeter latex strips were glued to the anterior, lateral, and medial margins of each bone (Figure 1). The objective was to create the need to apply a force of approximately 80 N on the fracture to proceed with the fracture reduction.

For the evaluation of the simulator, the process was divided into two stages and the research met the fundamental ethical and scientific requirements of Resolution No. 466/2012,13 in both. All participants signed the informed consent form, since the study was referred to the Research Ethics Committee of the Pontifical Catholic University of Paraná via Plataforma Brasil and obtained a favorable opinion (CAAE: 09525919.5.0000.0020).

The first stage of the simulator evaluation consisted of practical tests with orthopedic physicians at different levels of training. The goal was to obtain their opinion on the simulator’s ability to reproduce fracture reduction by their practical experience. To this end, a questionnaire designed by the researchers was applied, using a Likert psychometric scale. The participants answered about the simulator’s resemblance to reality, its external appearance, the traction force needed to perform the fracture reduction, and the reduction technique that could be performed on the simulator. Ten physicians participated in this stage, five were third-year orthopedic residents from the Cajuru University Hospital of the Pontifical Catholic University of Paraná, and five were orthopedic specialists from the same institution.

During the fracture reduction practice, the professionals were also evaluated using the OSATS form (Objective Structured Assessment of Technical Skill). Such form was initially developed to evaluate surgical techniques but was adapted for the evaluation of orthopedic skills and today is one of the most used instruments in the literature for simulator validation. This instrument evaluates five criteria and scores from zero to five points for each criterion, namely: respect for the model, knowledge, handling, operation flow, and operation time.^8^

In the second stage of the simulator evaluation, 16 medical students attended a theoretical class on pediatric forearm fractures and had a practical demonstration of the reduction technique performed on the simulator. Then, the students practiced the technique and the performance of the skills acquired with the activity was evaluated using the OSATS. Note that the form was applied by an orthopedic surgeon, not directly involved with the development of the simulator.

## RESULTS

The final cost of the project was 125 US dollars. The model built is of a pediatric arm and forearm, 35-cm long and weighing 900 g. It features an anterior medial opening to visualize and manipulate the fracture prior to the training.

**Figure 2:**
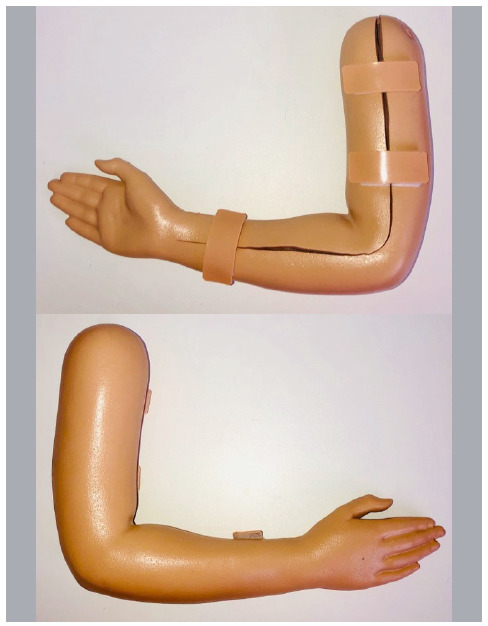
External and internal appearance of the orthopedic simulator.

The way it was developed, the simulator allows the training of reduction of some fracture types in different techniques, since the fracture fragments can be manipulated in a way to mimic greenstick fractures, complete, with or without deviation or rotations. The skin and soft parts of the simulator allow palpation, so that one can feel the bone landmarks as well as the unevenness generated by the fracture (Figure 2).

In the result of the physicians’ evaluation regarding the simulator’s similarity with reality. Eight of the 10 participants partially agreed with the statement “the simulator is quite realistic, matching with the reality of closed pediatric forearm fracture reduction,” and two of them totally agreed.

As for the external appearance, three of the interviewees stated that it is very similar, and seven of them said that the simulator is similar to the case of a real person.

Regarding the traction force required to perform the fracture reduction on the simulator, seven participants stated that it is similar to a real fracture. Two respondents considered the traction force not very similar, and both belong to the group of third-year orthopedic residents.

Regarding the reduction technique that could be performed in the simulator, seven participants said it was similar or very similar to the real thing. In this step, the participants have not received guidance on how to perform a specific technique; they were able to perform the technique they use the most in their day-to-day medical practice.

The participants’ overall performance during the reduction practice was obtained by the evaluation with the OSATS form. The results were analyzed using the mean ± standard deviation for each group. The results were: academics 14 ± 5.1 (5-21); 3rd year orthopedic residents 23.6 ± 1.34 (22-25); orthopedic specialists 24.5 ± 0.57 (24-25). The overall average score obtained by each group was proportional to their respective level of training. That is, the performance of medical students was the lowest and presented the highest standard deviation. In contrast, the more experienced groups had better and more uniform performances.

**Figure 3:**
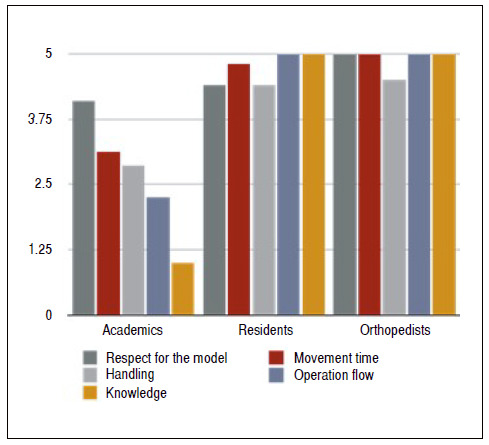
Graph of the performance of the participants in the specific criteria evaluated by the Objective Structured Assessment of Technical Skill form.

In analyzing the performance of the participants in the different criteria assessed by the OSATS, the criteria in which the students showed most difficult are the ones with the lowest scores: knowledge about the procedure and the flow of the operation, as shown in Figure 3. The students were very cautious when handling the simulator, as represented by the good scores obtained in the criterion of respect for the model (4.12 ± 1.45).

## DISCUSSION

The physicians agreed that the simulator performed well in manipulation and technique, and faithfully represents a real case. All participants agreed, to some degree, that the simulator is very realistic and matches the reality of closed reduction of pediatric forearm fractures. Among the criteria evaluated, external appearance was the best evaluated, while the traction force required for fracture reduction had the lowest evaluation.

As for the performance of the participants evaluated by the OSATS form, the overall average score obtained by each group was proportional to their respective level of training. The scholars’ mean scores in all evaluated criteria were the lowest. In other words, the simulator proved to be able to level out the participants according to different levels of training. It was indeed more difficult for the less experienced participants. If this were not the case, and the scores of the three groups were similar, it is likely that the stress force on the fracture and/or the reduction technique required would be so weak that an inexperienced person could easily perform the fracture reduction successfully.

The highest standard deviation perceived in the overall performance of the groups was that of the medical students. This fact objectifies what was seen in practice during the tests, that some students have a greater instinctive ease with the technique, although it is their first time practicing. Meanwhile, others had greater difficulty and insecurity to perform the reduction. These differences show the variety of profiles of the students, and how necessary it is for medical education to adapt to meet the different demands.

The validation of this simulator estimated the participants’ ability to reduce the proposed fracture; however, the quality of the reduction was not evaluated. Some papers in the literature used artificial radiopaque bones and control X-rays before and after reduction.^5^ ^),(^ ^6^ In these cases, bone alignment and residual deviations were compared to the results obtained through OSATS.

Our study aimed to develop a simulator with the purpose of being used for teaching medical students, as well as being the probable first contact with a closed reduction. Thus, the methodological and budgetary difficulties generated by the use of the control X-ray would not be justifiable. However, if the model is used for the purpose of training orthopedic specialists or residents, validations to estimate the quality of the applied closed reduction are necessary.

This skills practice in the preclinical setting allows students to maximize the learning opportunities presented to them in the clinical setting, build self-confidence, and contribute to improving the quality of care for their future patients. This is because it familiarizes general practitioners with the care of a patient with a fracture, and in this situation, to be able to identify, manipulate, and/or immobilize the patient and refer them to a specialized service, where they will receive the final treatment.

Validations that estimate the quality of the employed closed reduction are necessary if the model is meant to be used for the purpose of training orthopedic specialist physicians.

## CONCLUSION

A low-cost simulator was developed for teaching the technique of closed reduction of pediatric mid-third forearm fractures. The evaluation of the model was satisfactory and provides a good approximation of the basic orthopedic skill of manipulating and reducing a middle third forearm fracture. Thus, the model can be used as a teaching tool for training orthopedic residents and medical students.
